# AN AGENTIC PERSPECTIVE ON AGING PREPARATION

**DOI:** 10.1093/geroni/igad104.3010

**Published:** 2023-12-21

**Authors:** Yaeji Kim-Knauss, Frieder Lang

**Affiliations:** Friedrich-Alexander-Universität Erlangen-Nürnberg, Nuremberg, Bayern, Germany; Friedrich-Alexander-Universität Erlangen-Nürnberg, Nuremberg, Bayern, Germany

## Abstract

When imagining one’s future life in old age individuals may respond to an uncertain future with proactive and preparatory activity aiming to maximize possible rewards and minimize risks or costs. The current research applies an agentic perspective on aging preparation that emphasizes the role of individuals’ beliefs and attitudes in determining their engagement in actions. The present research aims to investigate the associations between action-related beliefs (perceived utility/risk) and situational cues (experience/appraisals of aging-related life challenges) with individuals’ engagement in aging preparation. Data comes from the “Ageing as Future” project, which includes multicultural (i.e., Germany, US, Hong Kong, Taiwan, Czechia), longitudinal (i.e., five measurement points spanning from 2012 to 2020), and lifespan data (i.e., aged 18 to 98). Results from a series of analyses indicate that perceiving more utilities, having experience in caregiving, and having health-related worries at the peak of the COVID-19 pandemic were associated with engagement in aging preparation. However, the magnitude of these associations varied depending on the time perspective and cultural context. Findings suggest that individuals engage in aging preparation to attain desirable or to avoid undesirable outcomes in the future based on personal values and belief systems. The dynamic interplay of personal belief systems with temporal and cultural contexts was also highlighted that differently shapes individual’s agentic engagement in aging preparation.

